# Lightweight Correlation Inference and Prediction of Asphalt Mixture Image Processing Technology Based on Dual-Tower Qwen

**DOI:** 10.3390/ma19214700

**Published:** 2026-11-04

**Authors:** Xunqian Xu, Cheng Zhou, Qian Xiao, Wenxuan Ge, Xu Wu, Shuyong Pan, Chen Zhang

**Affiliations:** 1 School of Transportation and Civil Engineering, Nantong University, Nantong 226019, China; xunqian_xu@ntu.edu.cn (X.X.); 2410310009@stmail.ntu.edu.cn (W.G.); ntu_panshuyong@163.com (S.P.); 2 School of Mechanical Engineering, Nantong University, Nantong 226019, China; zhou15283958510@163.com; 3 Research Institute of Highway, Ministry of Transport, Beijing 100088, China; xiaoqian202601@163.com; 4 Department of AIOps & IoT Research, Nantong Institute of Technology, Nantong 226002, China; wuxu8510@163.com

**Keywords:** asphalt mixture, literature mining, Qwen-STGF model, large language model, lightweight

## Abstract

To address the bottlenecks of low accuracy in cross-domain technological association recognition and difficulties in quantitative prediction of technological trends encountered when applying large language models to literature mining of asphalt mixture image processing technology, this paper proposes a multi-module collaborative dual-tower Qwen model (Qwen-STGF). A dataset is constructed from the academic literature in this interdisciplinary field. First, an improved dual-tower network is built by incorporating nonlinear activation functions and Dropout regularization, using the Qwen-1.8B-Chat large model as the shared semantic backbone to achieve unified mapping of the semantic features of keywords and titles. Second, learnable bias vectors are introduced to optimize the GraphSAGE network, thereby enhancing its capability for extracting spatial structural features. Meanwhile, a CNN-LSTM hybrid architecture is employed to construct a three-dimensional fused feature representation system encompassing semantic, spatial, and temporal dimensions, and a weighted random forest classifier with a class weight balancing strategy is adopted. Finally, a lightweight framework is developed for keyword co-occurrence prediction and quantitative technological-trend forecasting. The results demonstrate that compared with five types of traditional machine learning models, two types of classical graph neural network models, five native Chinese representation models, and four open-source large models of similar scale, the Qwen-STGF model achieves Precision, Recall, F1-score, AUC, and AUPR of 0.8673, 0.8162, 0.841, 0.8786, and 0.9322, respectively, among which AUC, AUPR, and F1-score rank as the best among all comparative models. This study reveals that keyword co-occurrence frequency can reflect the maturity of technology convergence to a certain extent, and the predicted trends correspond to the potential breakthrough prospects of relevant technologies. Three major future research directions are forecasted: establishing a unified mesoscopic feature parameter system, developing digital twin technology, and constructing reliable macro-mesoscopic performance correlation models. These directions provide technical support for asphalt mixture image processing.

## 1. Introduction

Asphalt pavement is the primary pavement structure for high-grade highways and urban roads. As the core material of asphalt pavement, the macroscopic service performance of asphalt mixture essentially stems from the mechanical response and evolution of its internal mesostructure under the coupled action of loads and environmental factors [1,2,3]. Therefore, accurate characterization of mesostructure has always been a core scientific issue in the field of road engineering. Image processing technology for asphalt mixtures [4,5], with its unique advantages of non-destructive testing, full-dimensional quantification and visual presentation, has become the core technical system for mesostructure characterization. With the rapid development of computer vision technology, the volume of the academic literature related to image processing technology for asphalt mixtures has increased exponentially. However, while the massive research achievements provide a rich data foundation for technological development, they also bring new challenges such as information overload, obscured technical evolution trajectory, and unpredictability of cutting-edge research directions. Traditional literature reading can no longer meet the needs of researchers to quickly grasp the overall technical picture, identify potential technical connections, and develop sound research agendas. Therefore, carrying out prospective research on image processing technology for asphalt mixtures has important practical value for promoting the development of this field.

Technology forecasting, a core approach to identifying disciplinary development directions, has undergone a gradual transition from traditional experience-driven paradigms to data-driven methodologies. Early technology forecasting predominantly relied on expert judgment [6], which yielded relatively reasonable results in data-scarce contexts. However, expert resources are limited in highly interdisciplinary domains such as road engineering and computer vision. In recent decades, the academic literature has emerged as the primary data source for technology development forecasting [7]. Conventional curve-fitting-based forecasting methods [8] can roughly estimate technology maturity timelines and project future trends based on current status. However, technological changes exhibit dynamic complexity, and the application of image processing technologies in road engineering is driven by multiple factors, including materials science and engineering demands, leading to limited predictive accuracy. Some researchers have proposed a patent citation analysis approach based on the Hawkes process [9], but this method is heavily reliant on citation network data. The application of deep learning approaches has substantially enhanced forecasting efficiency; however, these methods lack the integration of domain-specific expertise and thus exhibit limited domain adaptability [10,11]. Hybrid forecasting methodologies are increasingly becoming the dominant paradigm, exemplified by gap technology forecasting approaches that integrate clustering and Bayesian inference.

Currently, technology forecasting is primarily implemented through predictive models [12], with mainstream techniques focusing on Natural Language Processing (NLP) for textual content and link prediction for network structures. The former mostly involves unsupervised learning tasks such as topic identification [13] and semantic analysis [14], which can predict potential future topics from the perspective of technical content. However, it struggles to anticipate previously unseen terms, resulting in significant challenges in forecasting disruptive technologies [15]. The latter evaluates the probability of potential future node links using known network structures [16]. Early static link prediction models did not incorporate time as a critical input variable. Subsequent researchers constructed models using time window processing techniques, advancing the research on pseudo-dynamic technology forecasting [17]. Both traditional machine learning and deep learning methods have been extensively applied in link prediction [18].

In summary, existing technology forecasting models still face two core challenges when applied to image processing technology for asphalt mixtures. First, the accuracy of cross-domain technical association identification is insufficient. Second, it is difficult to make quantitative predictions of future technological development trends. In recent years, the development of deep learning and graph neural networks has provided new technical pathways to address these pain points. Siamese neural networks have been applied in fields such as recommendation systems and information retrieval. The CNN-LSTM hybrid structure can simultaneously capture long-term dependencies and local feature mutations in sequential data [19,20], and Farsi et al. [21] used this method to achieve short-term power load forecasting. GraphSAGE aggregates information by randomly sampling neighboring nodes [22,23,24]. However, using the above algorithms alone still suffers from problems such as a single feature dimension and insufficient semantic understanding of domain-specific terminology. Meanwhile, Large Language Models (LLMs) possess powerful semantic understanding and zero-shot generalization capabilities, which can compensate for the shortcomings of traditional machine learning at the semantic level, and their applications have been extensively studied [22,25,26]. Li et al. [27] implemented automated building load forecasting based on a multi-agent LLM framework. Sharma et al. [28] combined LLMs with YOLOv11 for lung cancer detection, verifying the feasibility and superiority of integrating LLMs with traditional algorithms. In addition, numerous studies have conducted technical association prediction based on International Patent Classification (IPC) co-occurrence relationships [15,29,30], providing new ideas for research in this field.

To address these limitations, this paper proposes a multi-module collaborative Qwen-STGF model targeting the core problems in literature mining of image processing technology for asphalt mixtures. First, an improved dual-tower semantic network integrated with the Qwen-1.8B-Chat large language model is proposed. By adopting Siamese fully connected branches and regularization strategies, the network realizes unified semantic feature mapping for keywords and paper titles, thereby remedying the limitation of traditional models in understanding the semantics of domain-specific terminology. Second, a co-occurrence intensity-weighted neighbor aggregation mechanism and learnable domain bias vectors are introduced into the weight-optimized GraphSAGE graph network. This enhancement strengthens the spatial structural feature extraction capability of the keyword co-occurrence network and distinguishes the semantic discrepancy between incidental co-occurrence and strong technical correlation. Finally, a three-dimensional (semantic–spatial–temporal) feature fusion framework is constructed. Coupled with a class-weight-balanced weighted random forest classifier, the framework constitutes a complete solution for keyword co-occurrence prediction and quantitative assessment of technology trends, enabling accurate identification of cross-domain technical correlations and quantitative analysis of development trajectories.

## 2. Model Architecture Design

This study aims to propose a widely applicable lightweight solution for small and medium-sized enterprises (SMEs) and individual researchers with limited large-scale computing resources. Large-parameter models generally rely on professional cloud GPU clusters to operate, whereas this study is conducted solely in the experimental environment of a single consumer-grade laptop. In light of the above considerations, the Qwen-1.8B-Chat model with 1.8 billion parameters, which can run stably on consumer-grade hardware, is selected. On this basis, a keyword co-occurrence prediction and technology trend forecasting system based on the Qwen-STGF model is constructed, forming an integrated technical solution covering literature data processing, phased multi-module training, lightweight online inference, and quantitative analysis of technology trends.

The Qwen-STGF model comprises five components: the Qwen dual-tower text semantic feature module, the GraphSAGE spatial graph feature module, the CNN-LSTM temporal evolution feature extraction module, the multi-feature fusion module, and the weighted random forest classification module. With complementary functionalities and collaborative operation, all modules constitute a progressive technical logic: semantic foundation representation, spatial structure modeling, temporal evolution deduction, and fusion decision output.

The Qwen dual-tower text semantic feature module generates standardized semantic features for nodes and edges, which serve as high-quality initial feature inputs for the subsequent graph network module. The GraphSAGE spatial graph feature module extracts topological structural features of technical correlations based on annual keyword co-occurrence networks. The annually generated spatial features are stacked along the temporal dimension to construct the input sequence of the temporal feature module. The CNN-LSTM temporal evolution feature extraction module models the multi-year spatial feature sequence to capture the dynamic evolution patterns of the technical correlation network.

**Figure 1:**
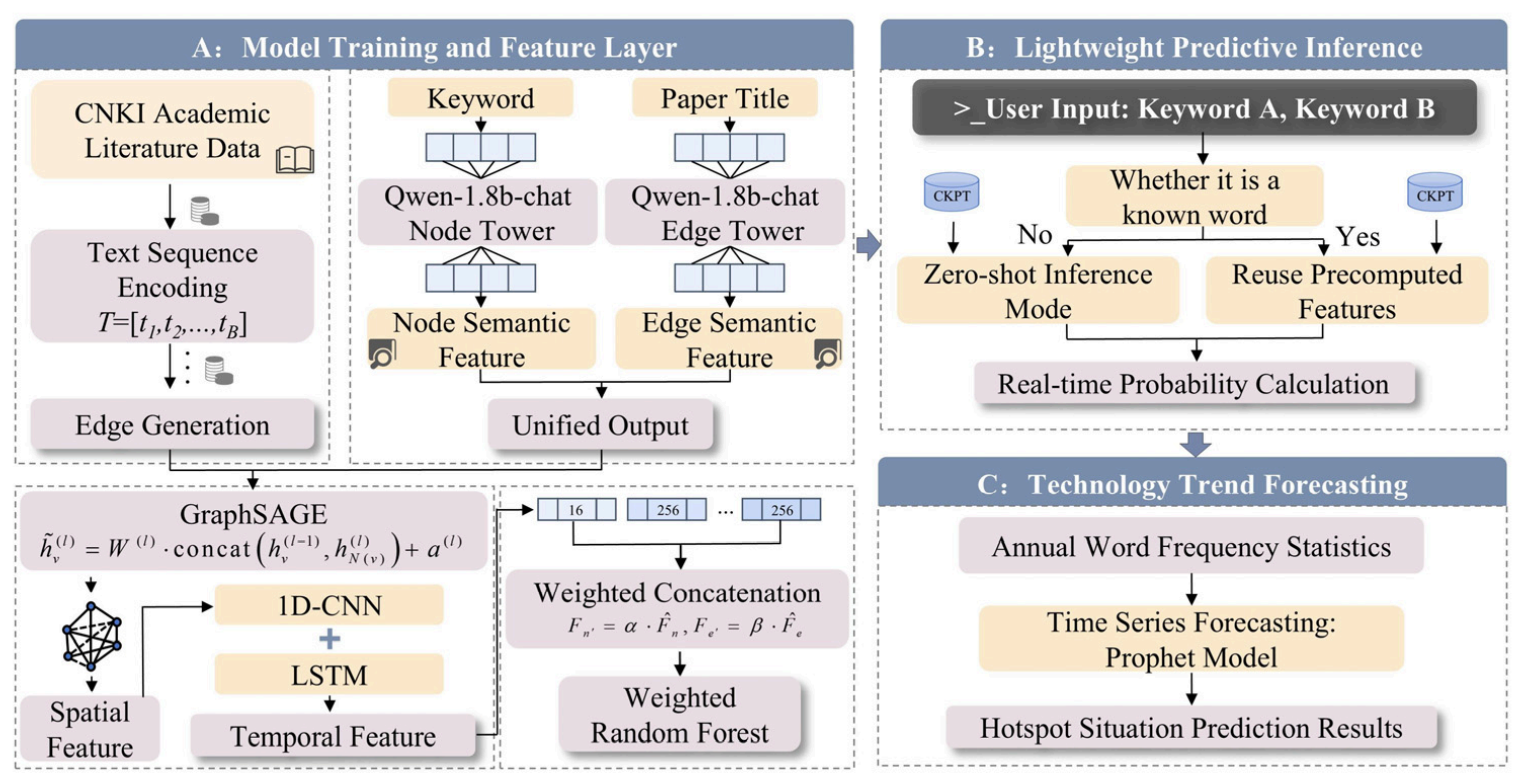
Overall architecture of the model.

Following weighted fusion, the three categories of features are fed into the weighted random forest classifier to output keyword association probabilities. Technology trend forecasting is then implemented by integrating temporal word frequency data. The overall architecture of the proposed model is illustrated in Figure 1.

### 2.1. Qwen-Two-Tower Text Semantic Feature Extraction Module

Global text semantic features are extracted through the unified Qwen-1.8B-Chat large model backbone. Then, independent Siamese fully connected branches are designed for keyword nodes and literature title edges to complete the mapping from high-dimensional semantic features to task-adaptive low-dimensional dense features. Finally, normalization is performed to eliminate dimensionality effects, outputting standardized text semantic features that can be directly fused with spatial and temporal features.

#### 2.1.1. Text Sequence Encoding

First, the module performs tokenization, padding to a fixed length, and mask construction on the input unstructured text (keywords/titles), converting the text into numerical tensors processable by the LLM. The mathematical formulation is given by the following:

For a batch text list *T* = [*t*_1_,*t*_2_,…,*t_B_*] (where *B* is the batch size), the unequal-length input index sequences $I_{i}\in N^{L_{i}}$ (where *L_i_* is the length of the *i*-th text) are obtained after Tokenizer encoding, and are unified to a fixed length *L*_max_ (length *L*_max_ = 16 for keywords, length *L*_max_ = 64 for titles):

(1) $$I_{i}^{\prime }=\left\{\begin{array}{ll} I_{i}[0:L_{\max}] & L_{i}>L_{\max}\\ \mathrm{concat}(I_{i},{\left[\mathrm{pad}\_\mathrm{id}\right]}_{1\times (L_{\max}-L_{i})}) & L_{i}<L_{\max}\\ I_{i} & L_{i}=L_{\max} \end{array}\right.$$

where $I_{i}^{\prime }$ denotes the output sequence after length normalization; *I_i_* denotes the *i*-th original input sequence; *L_i_* is the original length of the *i*-th sequence; *L*_max_ is the preset maximum sequence length threshold; *concat*(⋅) represents the sequence concatenation operation; ${\left[\mathrm{pad}\_\mathrm{id}\right]}_{1\times (L_{\max}-L_{i})}$ denotes the padding vector with length *L*_max_ − *L_i_*; *pad*_*id* is the preset padding token used to mark invalid tokens.

Finally, the equal-length input tensor $I\in N^{B\times L_{\max}}$ is generated, and the attention mask tensor is

(2) $$M_{i}[j]=\left\{\begin{array}{l} \begin{array}{cc} 1 & j<L_{i} \end{array}\\ \begin{array}{cc} 0 & j\geq L_{i} \end{array} \end{array}\right.$$

#### 2.1.2. Qwen-1.8B-Chat Basic Semantic Extraction

The encoded tensor is fed into the frozen Qwen-1.8B-Chat large model to extract global high-dimensional semantic features of the text. The mathematical operation corresponding to the semantic core of this module is as follows:

During feature extraction, all LLM encoders were executed in evaluation mode under the torch.no_grad() context, with parameters completely frozen to reuse only pre-trained weights. All LLM baselines followed this identical protocol to ensure a fair comparison.

Model forward propagation: Input *I* and *M* into the Transformer encoder of Qwen-1.8B-Chat. After computation through L_trans_ layers of self-attention and feed-forward networks, the hidden states $H^{1},H^{2},\ldots ,H^{L_{\mathrm{trans}}}$ of each layer are output. The hidden state $H^{L_{\mathrm{trans}}}\in R^{B\times L\times D_{q}}$ of the L_trans_-th layer (the last layer) contains the most abstract textual semantic information.

The feature at the 0-th position of the last-layer hidden state is selected as the global semantic representation of a single text. The formula is

(3) $$F_{q}=H^{L_{\mathrm{trans}}}[:,0,:]\in R^{B\times D_{q}}$$

where *F_q_* is the basic semantic feature of Qwen; $H^{L_{\mathrm{trans}}}$ is the hidden state (*B* × *L*_max_ × *D_q_*) of the last Transformer layer in Qwen-1.8B-Chat; and *D_q_* = 2048 denotes the fixed hidden dimension of Qwen-1.8B-Chat.

#### 2.1.3. Siamese Two-Tower Feature Mapping

Considering the differences in textual characteristics between keyword nodes and literature title edges, a structurally independent and weight-unshared Siamese dual-branch fully connected network is designed. This network maps 2048-dimensional high-dimensional semantic features into low-dimensional dense features of code-specified dimensions respectively. Meanwhile, nonlinear activation functions and Dropout regularization are introduced to enhance feature representation capability and prevent overfitting. The two branches share the same mathematical principles.

(1) Keyword Node Branch (mapping to 16-dimensional node features):

This branch performs dimensionality reduction mapping of *D_q_* → *D_n_*_1_ → *D_n_*, formulated as

(4) $$F_{n1}=\mathrm{ReLU}\left(F_{q}\cdot W_{n1}+b_{n1}\right)\in R^{B\times D_{n1}}$$

(5) $$F_{n1}^{\prime }=\mathrm{Dropout}\left(F_{n1},p=0.1\right)\in R^{B\times D_{n1}}$$

(6) $$F_{n}=F_{n1}^{\prime }\cdot W_{n2}+b_{n2}\in R^{B\times D_{n}}$$

where $W_{n1}\in R^{D_{q}\times D_{n1}}$ and $b_{n1}\in R^{D_{n1}}$ are the weight and bias of the first fully connected layer; $W_{n2}\in R^{D_{n1}\times D_{n}}$ and $b_{n2}\in R^{D_{n}}$ are the weight and bias of the second fully connected layer; and *ReLU* is the nonlinear activation function.

(2) Literature Title Edge Branch (mapping to 256-dimensional edge features):

This branch performs dimensionality reduction mapping of *D_q_* → *D_e_*_1_ → *D_e_*, formulated as

(7) $$F_{e1}=\mathrm{ReLU}\left(F_{q}\cdot W_{e1}+b_{e1}\right)\in R^{B\times D_{e1}}$$

(8) $$F_{e1}^{\prime }=\mathrm{Dropout}\left(F_{e1},p=0.1\right)\in R^{B\times D_{e1}}$$

(9) $$F_{e}=F_{e1}^{\prime }\cdot W_{e2}+b_{e2}\in R^{B\times D_{e}}$$

where $W_{e1}\in R^{D_{q}\times D_{e1}}$ and $b_{e1}\in R^{D_{e1}}$ are the weight and bias of the first fully connected layer in the title branch; $W_{e2}\in R^{D_{e1}\times D_{e}}$ and $b_{e2}\in R^{D_{e}}$ are the weight and bias of the second fully connected layer in the title branch.

Dropout is a random dropout regularization that randomly sets some feature values to zero with a probability in the range (0,1) to avoid overfitting of the branch. The activation function and regularization strategy are consistent with those of the node branch to ensure the consistency of feature transformation.

The two branches of the Siamese twin-tower network share the semantic backbone of Qwen-1.8B-Chat, ensuring that the semantic features of keywords and titles are mapped to the same feature space. Meanwhile, the independent fully connected weights can adapt to the textual characteristics of both, thus avoiding feature interference.

#### 2.1.4. L2 Normalization

To eliminate the dimension differences among different text features and ensure the comparability of Euclidean distances and fusion effectiveness with GraphSAGE spatial features and CNN-LSTM temporal features in subsequent steps, L2 normalization [31,32] is applied to the low-dimensional features output by the two-tower network. The normalized feature formulas for keyword nodes and document title edges are given by

(10) $${\hat{F}}_{n}=\frac{F_{n}}{\|F_{n}{\|}_{2}}=\frac{F_{n}}{\sqrt{\sum_{d=1}^{D_{n}}F_{n,:,d}^{2}}}\in R^{B\times D_{n}}$$

(11) $${\hat{F}}_{e}=\frac{F_{e}}{\|F_{e}{\|}_{2}}=\frac{F_{e}}{\sqrt{\sum_{d=1}^{D_{e}}F_{e,:,d}^{2}}}\in R^{B\times D_{e}}$$

where ‖⋅‖_2_ denotes the L2 norm. After normalization, the L2 norm of all features is 1, and the feature vectors lie on the unit hypersphere. This not only ensures feature standardization but also preserves the original semantic differences among features.

### 2.2. GraphSAGE Spatial Graph Feature Extraction Module

The standard GraphSAGE employs an equal-weight neighbor aggregation mechanism, which fails to effectively differentiate the semantic discrepancy between incidental co-occurrence and co-occurrence with strong technical correlation in keyword co-occurrence networks. To address this limitation, a weighted GraphSAGE model based on co-occurrence intensity is proposed. The node feature update rules are optimized through two strategies, namely weighted neighbor aggregation and learnable domain bias vectors, thereby enhancing the spatial topological feature extraction capability of the keyword co-occurrence network.

#### 2.2.1. SAGEConv Graph Convolutional Layer

The node feature update formula of the *l*-th GraphSAGE layer:

First, weighted aggregation is performed on the neighbor features of the target node *v*, with the aggregation weights being the co-occurrence intensities of the corresponding edges:

(12) $$h_{N(v)}^{\left(l\right)}=\frac{\sum_{u\in N(v)}w_{\mathrm{uv}}\cdot h_{u}^{\left(l-1\right)}}{\sum_{u\in N(v)}w_{\mathrm{uv}}}$$

where *N*(*v*) is the first-order neighbor set of node *v*; *w_uv_* is the weight of the edge connecting nodes *u* and *v*; $h_{u}^{\left(l-1\right)}$ is the feature vector of node *u* at the (*l* − 1)-th layer; and $h_{N(v)}^{\left(l\right)}$ is the neighbor-aggregated feature vector of node *v*.

Rather than directly employing raw counts, the edge co-occurrence weight *w_uv_* is computed via logarithmic compression and normalization to ensure cross-year comparability and prevent high-frequency pairs from dominating the aggregation, which is formulated as

(13) $$w_{\mathrm{uv}}^{\left(t\right)}=\frac{\log(1+c_{\mathrm{uv}}^{\left(t\right)})}{\log(1+N_{t})+\lambda \left[\log(1+\deg^{\left(t\right)}(u))+\log(1+\deg^{\left(t\right)}(v))\right]}$$

where $c_{\mathrm{uv}}^{\left(t\right)}$ denotes the cumulative co-occurrence frequency of keywords *u* and *v* within the same publication up to year *t*; *N_t_* represents the total number of co-occurrence events in year *t*; deg^(*t*)^(*u*) is the degree of node *u* in year *t*; and *λ* serves as the degree normalization coefficient.

Specifically, the logarithmic transformation compresses the dynamic range of high-frequency co-occurrence pairs; division by the annual total *N_t_* eliminates discrepancies arising from varying publication volumes across years, thereby ensuring cross-year comparability; normalization by node degrees mitigates the dominance of hub nodes and their associated high-weight edges, which aligns with the frequency normalization rationale of TF-IDF and Pointwise Mutual Information (PMI).

Subsequently, the self-features of the target node are concatenated with the aggregated neighbor features, and the updated node features are obtained through linear transformation. A domain-specific bias vector is introduced to better align the output results with the engineering scenario characteristics of asphalt mixture image processing technology:

(14) $${\tilde{h}}_{v}^{\left(l\right)}=W^{\left(l\right)}\cdot \mathrm{concat}\left(h_{v}^{\left(l-1\right)},h_{N(v)}^{\left(l\right)}\right)+a^{\left(l\right)}$$

where $h_{v}^{\left(l-1\right)}$ is the feature vector of node *v* at the (*l* − 1)-th layer; concat(⋅) denotes the vector concatenation operation; *W*^(*l*)^ is the learnable weight matrix of the *l*-th graph convolution layer; *a*^(*l*)^ is the learnable bias vector of the *l*-th graph convolution layer; and ${\tilde{h}}_{v}^{\left(l\right)}$ is the intermediate feature vector after linear transformation.

To improve the generalization ability and training stability of the model, this paper introduces the ReLU activation function and Dropout regularization in the intermediate layers, and applies batch normalization in the final layer. Finally, L2 normalization is performed on the output features to scale them to unit length:

(15) $$h_{v}^{\left(l\right)}=\left\{\begin{array}{ll} h_{\mathrm{drop}}\left(\sigma \left({\tilde{h}}_{v}^{\left(l\right)}\right)\right), & l<L\\ h_{\mathrm{bn}}\left({\tilde{h}}_{v}^{\left(l\right)}\right), & l=L \end{array}\right.$$

(16) $$h_{v}^{\mathrm{out}}=\frac{h_{v}^{\left(L\right)}}{{\left\|h_{v}^{\left(L\right)}\right\|}_{2}}$$

where *σ*(⋅) is the nonlinear activation function, i.e., ReLU; $h_{v}^{\left(l\right)}$ is the output feature vector of node *v* after the *l*-th graph convolution; *h_drop_* is the output feature vector after the Dropout operation; *h_bn_* is the output feature after batch normalization transformation; $h_{v}^{\mathrm{out}}$ is the final output feature vector of the entire GraphSAGE module; and $h_{v}^{\left(L\right)}$ is the node feature vector at the *L*-th (final) layer.

#### 2.2.2. InfoNCE Loss

The InfoNCE loss from the field of contrastive learning [33,34] is adopted as the training objective of GraphSAGE. For each keyword node acting as an anchor, the model is trained to minimize its feature space distance to co-occurring keyword nodes (positive samples) and maximize its distance to non-co-occurring keyword nodes (negative samples). The complete mathematical formulation is given by

(17) $$L_{\mathrm{InfoNCE}}=-\frac{1}{N}\sum_{i=1}^{N}\log\frac{e^{\mathrm{sim}(h_{i},h_{i}^{+})/\tau }}{\sum_{j=1,j\neq i}^{N}e^{\mathrm{sim}(h_{i},h_{j}^{-})/\tau }}$$

where *h_i_* denotes the feature vector of the anchor sample; $h_{i}^{+}$ and $h_{j}^{-}$ represent the positive and negative samples corresponding to the anchor, respectively; *sim*(⋅) denotes the similarity function, implemented by cosine similarity $\mathrm{sim}(u,v)=\frac{u^{T}v}{{∥u∥}_{2}{∥v∥}_{2}}$ [35]; *τ* is the temperature coefficient; *N* is the total number of keyword nodes.

### 2.3. Multi-Feature Fusion and Weighted Random Forest Classification Module

#### 2.3.1. Feature Fusion

Building on the aforementioned static text semantic features from the Qwen dual-tower module, spatial topological features from the GraphSAGE module, and temporal evolution features from the CNN-LSTM module, a three-dimensional (semantic–spatial–temporal) feature fusion framework is established. A modal weighting strategy is introduced to enhance the contribution of high-importance modal features. Coupled with a class-weight-balanced weighted random forest classifier, this framework forms a complete solution for keyword co-occurrence prediction and quantitative assessment of technology trends.

Modal weights are determined via parameter sensitivity analysis. Taking Precision, Recall, and AUPR as optimization objectives, the text feature weight of keyword nodes and the text feature weight of title edges are set as tunable parameters. As verified by the analysis, the weight coefficient of keyword node text features is finalized at 3.6, and that of title edge text features is finalized at 2.95, to emphasize modal information with higher contribution. The corresponding mathematical formulation is presented as follows:

(18) $$F_{n^{\prime }}=\alpha \cdot {\hat{F}}_{n},F_{e^{\prime }}=\beta \cdot {\hat{F}}_{e}$$

(19) $$F_{\mathrm{node}}=\mathrm{concat}\left(F_{n^{\prime }},F_{s},F_{t}\right)\in R^{B\times \left(D_{n}+D_{s}+D_{t}\right)}$$

(20) $$F_{\mathrm{fused}}=\mathrm{concat}\left(F_{\mathrm{node},1},F_{\mathrm{node},2},F_{e^{\prime }}\right)\in R^{B\times \left(2D_{\mathrm{node}}+D_{e}\right)}$$

where ${\hat{F}}_{n}$ denotes the original node features of the graph; ${\hat{F}}_{e}$ denotes the original edge features of the graph; $F_{n^{\prime }}$ denotes the scaled node features of the graph; $F_{e^{\prime }}$ denotes the scaled edge features of the graph; *α*, *β* are the fixed scaling coefficients for features; *F_t_* denotes the static attribute features of nodes; *F_s_* denotes the temporal dynamic features of nodes; *F_node_* denotes the node-level multimodal fused features, where *D_node_* = *D_n_* + *D_s_* + *D_t_*; and *F_fused_* denotes the final multi-source fused features, where *D_fused_* = 2*D_node_* + *D_e_*.

The concrete definitions and dimensional configurations of the aforementioned features are specified as follows: the static keyword semantic feature is 16-dimensional, while both the spatial structural feature (*F_s_*) and the temporal evolutionary feature (*F_t_*) are 256-dimensional, yielding a node-level multimodal fused representation of 16 + 256 + 256 = 528 dimensions. The keyword-pair-level feature is constructed by concatenating the fused vectors of both endpoint nodes (528 dimensions each) with the edge title semantic feature (256 dimensions), resulting in a total dimensionality of 528 × 2 + 256 = 1312. Notably, rather than performing explicit pairwise temporal pooling, the node-level temporal feature *F_t_* is integrated into the pair-level representation via direct bilateral concatenation of the complete node vectors (*u* and *v*), enabling the downstream classifier to implicitly capture nonlinear interactions between the two technical entities.

#### 2.3.2. Weighted Random Forest Classification

Taking the multi-dimensional fused features as input, a weighted random forest classifier is constructed to complete the prediction of keyword association relationships. The decision tree splitting in the random forest is based on the Gini coefficient [36], as follows:

(21) $$\mathrm{Gini}(D)=1-\sum_{k=1}^{2}p_{k}^{2}$$

where *p_k_* is the proportion of the *k*-th class (positive/negative samples) in the sample set *D*.

### 2.4. Overview of Lightweight Association Reasoning and Technology Trend Prediction Algorithm

This study constructs a keyword co-occurrence prediction and technology trend analysis system oriented to asphalt mixture image processing technology. Given that individual researchers generally use keywords as the core analysis unit when conducting judgment on technological development trends, the input form of the lightweight association reasoning and technology trend prediction modules is set as keyword pairs. As illustrated in Algorithm 1.
**Algorithm 1:** Qwen-STGF academic keyword association prediction and trend reasoning.1. **Input:** User-input keyword pair *K_pair_*.2. **Initialization:** Precomputed feature library *F_pre_* extracted by the model, Qwen-STGF model weights *W_model_*, temporal word frequency database *F_time_*; inference mode *mode*, association probability *P* = 0, trend prediction result *T* = *ϕ*, final output result *R* = *ϕ*, feature vector *f* = *ϕ*.3. **Step 1: Lexical Matching Verification**4.  *is*_*precomputed* ← *checkVocabulary*(*K_pair_*,*F_pre_*)//Verify whether the keywords are present in the precomputed feature library.5. **Step 2: Feature Extraction**6.  **if** *is*_*precomputed*==*True* **then**7.    *f* ← *getPrecomputedFeature*(*K_pair_*,*F_pre_*)//Reuse the precomputed feature vector.8.  **else**9.    *f* ← *QwenSTGF*.*extractFeature*(*K_pair_*,*W_model_*)//Perform zero-shot real-time extraction of semantic features.10.  **end if**11. **Step 3: Keyword Association Probability Calculation**12.    *P* ← *QwenSTGF*.*predictCorrelation*(*f*,*W_model_*)//Output the association probability of keyword pairs.13. **Step 4: Keyword Temporal Trend Prediction**14.  **if** *mode*=="*trend*" **then**15.    *T* ← *predictTimeSeries*(*K_pair_*,*F_time_*,*T*)//Predict the word frequency change trends over the next 5 years.16.  **end if**17. **Step 5: Result Output**18.    *R* ← *combineResult*(*K_pair_*,*P*)//Integrate the association probability results.19.  **return** *R*

## 3. Experiments

### 3.1. Data Preprocessing

The China National Knowledge Infrastructure (CNKI) database is adopted as the literature data source, and relevant Chinese publications in the interdisciplinary field of asphalt mixture image processing are retrieved to construct the research dataset.

Retrieval strategy: A multi-field combined retrieval method covering subject, keywords, and abstract is employed. The retrieval formula is defined based on three CNKI search fields: SU (Subject), KY (Keywords), and AB (Abstract). The specific expression is SU = (asphalt mixture AND image processing) OR KY = (asphalt mixture AND image processing) OR AB = (asphalt mixture AND image processing). The literature types include Chinese core journals, journals indexed in the Chinese Science Citation Database (CSCD), journals indexed in the Chinese Social Sciences Citation Index (CSSCI), and master’s and doctoral dissertations. The publication period is from 1 January 2016 to 31 December 2025.

Inclusion criteria: Academic publications focusing on image processing, quantitative analysis, image segmentation and recognition of asphalt mixture mesostructure, with complete keywords and publication year information.

Exclusion criteria: Non-academic documents such as conference notices, book reviews, and calls for papers; publications with duplicate publication and highly overlapping content; and documents with missing core metadata.

A total of 778 relevant publications were obtained through preliminary retrieval. After duplication removal and screening, 768 eligible samples are retained, with core metadata including keywords, paper titles, and publication years. This retrieval strategy covers core journals and dissertation outputs in the field and can comprehensively reflect the current research status of asphalt mixture image processing technology in China.

Keyword splitting and node construction are performed on the collected literature. Keywords of each publication are split with semicolons as delimiters, and 1903 unique keyword nodes are finally generated. A set of positive and negative sample edges is constructed. Positive sample edges are generated based on keyword co-occurrence relationships: non-repetitive pairwise combinations of keywords within each publication are generated to form co-occurring keyword pairs. Each keyword pair corresponds to one positive sample edge assigned a label of 1 (indicating that technological convergence has occurred). A total of 8562 positive sample edges are obtained.

Negative sample edges are generated via a constrained random sampling method, with the ratio of negative to positive sample edges set at 0.625:1 (i.e., the number of negative sample edges is 0.625 times that of positive sample edges). To avoid cross-temporal label leakage and reduce the probability of mislabeling highly semantically relevant keyword pairs that have not yet co-occurred as negative samples, the sampling process abides by two constraints:

All negative samples are generated from the keyword set of the corresponding year to ensure they share the same temporal dimension as the positive samples. Keyword pairs with existing co-occurrence relationships in the sampling year and all prior years are excluded.

Because a negative keyword pair has never co-occurred, no authentic title embodies it, and assigning random titles would make edge text a proxy label, as positive titles contain both endpoints whereas random titles usually do not. To retain authentic titles while closing this leakage, we use a leakage-controlled protocol: (1) For positive edges, the authentic title is used—when several papers contain the same pair, one title is chosen by a fixed seed, and edges are grouped by paper so no title crosses training and test folds. (2) For negative edges, a counterfactual title is generated by the frozen Qwen under a fixed prompt, constrained to contain both endpoints and to match the CNKI title style. The heuristic “both keywords appear in the title” therefore holds for virtually all positive and negative instances and is non-discriminative.

Finally, 5353 negative sample edges are generated, each assigned a label of 0 (indicating that technological convergence has not occurred as of the corresponding year). The total number of positive and negative sample edges reaches 13,915. Meanwhile, the label definition is clarified: in this task, negative samples only represent the absence of historical co-occurrence and do not equate to the impossibility of future technological convergence. The association probability output by the model characterizes the latent potential of technological convergence.

The base positive rate of this dataset is 8562/13,915 = 0.6153. Under this class distribution, the expected AUPR baseline for both a majority-class classifier (which persistently predicts positive) and a random ranking classifier is identically 0.6153. The AUPR of 0.9322 achieved by our proposed model represents an absolute improvement of 0.3169 over this baseline, demonstrating that its superior discriminative performance is not an artifact of the class ratio. Positive instances outnumber negative ones primarily to avoid introducing substantial false negatives, which would inevitably occur if all unobserved node pairs were exhaustively enumerated across the complete graph; hence, a controlled negative sampling strategy was adopted to constrain the negative sample size to 0.625 times that of the positive instances.

### 3.2. Evaluation Metrics

Considering the extremely imbalanced characteristics of data in asphalt mixture image processing technology, five comprehensive evaluation metrics are selected, as shown in Table 1.

### 3.3. Experimental Parameter Settings

#### 3.3.1. Core Modules and Training Configuration

All experiments are implemented based on the PyTorch 2.1.0 framework and conducted in a hybrid configuration environment adopting a GPU-prioritized and CPU-compatible strategy. The parameter configurations are listed as follows:

The batch size is set to 16; the feature dimension of keyword nodes is 16, and the edge feature dimension of the literature titles is 256; the learning rate is 0.001, at which the model achieves the optimal balance between convergence speed and stability. The GraphSAGE spatial feature extraction module adopts a 3-layer network structure, with a hidden layer dimension of 256 and a neighbor sampling size of 15. The CNN-LSTM feature extraction module contains three LSTM layers with a hidden dimension of 512. The convolution kernel size is set to 3, and the number of output channels is 512. The number of decision trees is set to 350, the maximum depth is 24, and the minimum number of samples required for splitting is 2. Meanwhile, positive samples are assigned a weight of 2 and negative samples a weight of 1. The feature selection strategy adopts the log2 criterion to limit the number of features and reduce the risk of overfitting.

Assigning a double loss weight to positive instances reflects a deliberate cost-sensitive learning configuration: in prospective technology discovery scenarios, the penalty of overlooking an authentic emerging technological convergence (false negative) substantially outweighs the experimental effort of filtering false alarms (false positive); therefore, moderately upweighting positive instances serves to prioritize recall. This weighting scheme was finalized as part of our optimal parameter configuration (M15). Furthermore, because both AUC and AUPR are threshold-independent global ranking metrics governed by continuous predicted probabilities, they evaluate the relative scoring order rather than discrete decision boundaries, systematically precluding any artificial metric inflation induced by class reweighting.

#### 3.3.2. Parameter Sensitivity Analysis

**Figure 2:**
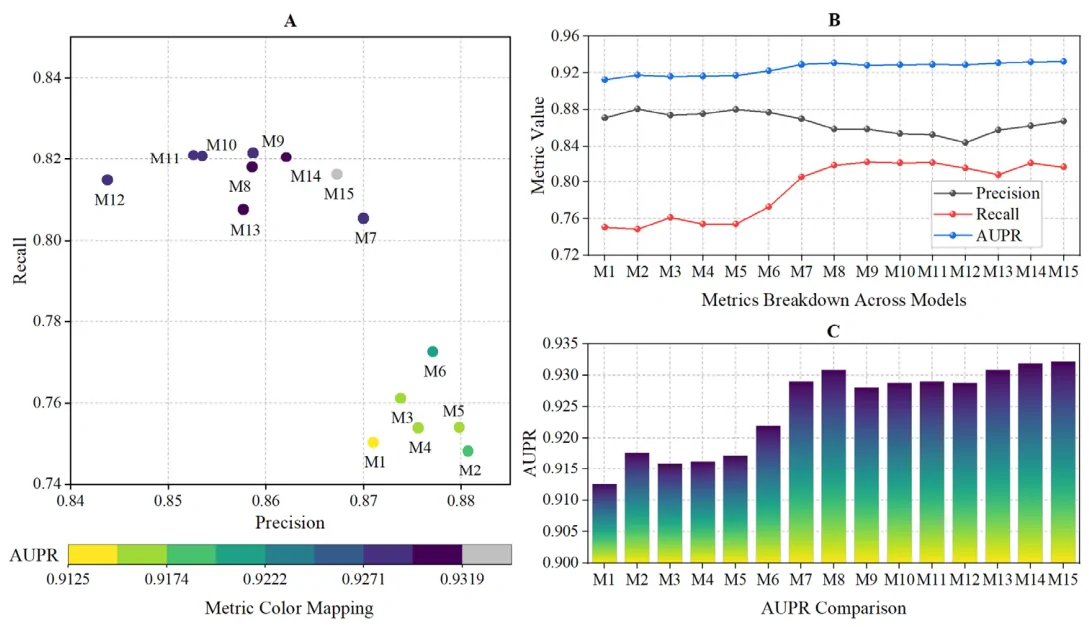
Model performance under different configurations. (**A**) Precision–Recall Scatter Plot: AUPR Values Mapped to Metric Color Scale; (**B**) Metrics Breakdown Across Models; (**C**) AUPR Comparison.

To evaluate the influence of hyperparameters on model performance, the control variable method is adopted to conduct parameter sensitivity experiments, with Precision, Recall and AUPR used as evaluation metrics. Different parameter configurations are listed in Table 2, and the model performance under various configurations is illustrated in Figure 2.

It can be seen from the results in Table 2 and Figure 2 that although models M1–M6 are slightly higher than M15 in Precision, the performances in Recall are significantly lower. Compared with model M8, its Recall (0.8216) and AUPR (0.9281) are close to those of M15, while M15 shows a greater advantage in Precision (0.8673). Through multiple rounds of parameter tuning and performance optimization, model M15 achieves the optimal balance among the three core indicators of Precision, Recall and AUPR, making it the configuration with the most balanced comprehensive performance in this experiment.

### 3.4. Experimental Result Analysis

#### 3.4.1. Baseline Model Comparison

**Figure 3:**
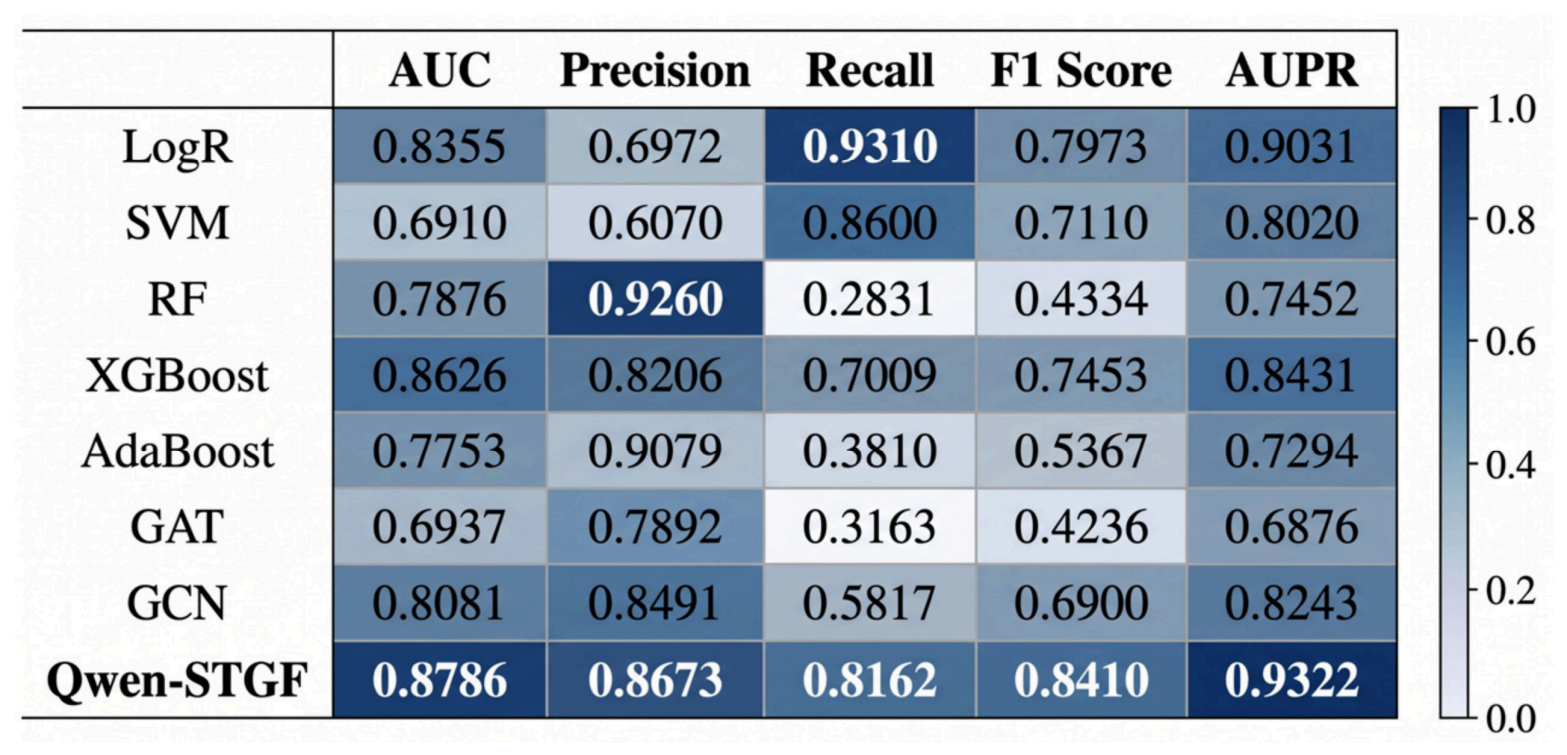
Model performance comparison.

To verify the effectiveness of the Qwen-STGF model, representative baseline models are selected for comparison. First, traditional machine learning algorithms, including Logistic Regression (LogR) [42], Support Vector Machine (SVM) [43], Random Forest (RF) [44], XGBoost [45], and AdaBoost [46]. Second, current mainstream advanced graph learning models include Graph Attention Network (GAT) [47] and Graph Convolutional Network (GCN) [48,49]. Tenfold stratified cross-validation is adopted to ensure the robustness of the results, and experiments are repeated, with the average value taken. The performance comparison between the Qwen-STGF model and baseline models is shown in Figure 3.

The aforementioned 10-fold cross-validation primarily evaluates the model’s capability in identifying synchronous technological associations. To prevent potential data leakage caused by keyword pairs originating from the same publication simultaneously appearing in both the training and test sets, a group-by-paper cross-validation scheme is adopted based on publication identifiers. Furthermore, to assess the model’s prospective inference capability on newly emerging co-occurrences, a strictly time-sliced link prediction protocol is supplemented, wherein historical data (year ≤ *t*) are utilized for training while future data (year ≥ *t* + 1) are withheld for testing (train ≤ *t*, test ≥ *t* + 1).

The Qwen-STGF model achieves the optimal performance among all models on three key metrics: AUC, AUPR and F1 Score, with the corresponding values reaching 0.8786, 0.9322 and 0.8410 respectively. Among them, the AUC is 1.6 percentage points higher than that of the suboptimal XGBoost model, the AUPR is 2.91 percentage points higher than that of the suboptimal LogR model, and the F1-score is 4.37 percentage points higher than that of the suboptimal LogR model. Meanwhile, the precision of the Qwen-STGF model reaches 0.8673, which is only lower than that of RF and AdaBoost; the recall reaches 0.8162, which is only lower than that of LogR and SVM. Compared with RF and AdaBoost, which have high precision but recall rates below 0.4, the recall of Qwen-STGF is improved by 53.31 and 43.52 percentage points respectively. Compared with LogR and SVM, which have high recall rates but precision rates below 0.7, the precision of Qwen-STGF is increased by 17.01 and 26.03 percentage points respectively, solving the imbalance deficiency of traditional baseline models. In addition, on the AUPR metric, Qwen-STGF is the only model that breaks through 0.93. It outperforms LogR (the best-performing model in traditional machine learning) by 2.91 percentage points and GCN (the best-performing model in graph learning) by 10.79 percentage points, which fully verifies the generalization ability and robustness of the model on imbalanced datasets.

To comprehensively evaluate the performance advantages of the proposed Qwen-STGF framework in link prediction tasks over the Chinese academic literature knowledge graphs, five representative Chinese text representation models were introduced as supplementary baselines, spanning two mainstream technical paradigms: Chinese pre-trained encoders (BERT-base-Chinese, Chinese-RoBERTa-wwm-ext, and ERNIE-3.0-Base-ZH) and Chinese sentence embedding methods (BGE-small-zh-v1.5 and Text2Vec-Base-Chinese). The performance comparison between Chinese baseline models is shown in Table 3.

As evidenced in Table 3, all Chinese baseline models exhibit a consistent performance pattern characterized by high recall and low precision: recall scores across all baselines exceed 0.98, with Chinese-RoBERTa-wwm-ext and BGE-small-zh-v1.5 reaching 0.9958 and 0.9956, respectively, indicating that purely semantic encoding models tend to indiscriminately classify candidate keyword pairs as associated. However, their precision values remain constrained within the 0.61–0.64 range—with the top-performing ERNIE-3.0-Base-ZH attaining merely 0.6342—reflecting a substantial false-positive rate in link prediction. Constrained by this severe imbalance between precision and recall, baseline F1-scores fluctuate narrowly between 0.7635 and 0.7729, demonstrating limited overall discriminative capacity. Regarding global ranking metrics, baseline AUC values range from 0.8323 to 0.8515, while AUPRs span 0.9030 to 0.9142; among them, Text2Vec-Base-Chinese achieves the strongest baseline AUC and AUPR, benefiting from specialized pre-training for Chinese semantic similarity tasks. This behavior reveals that unaugmented semantic encodings, lacking structural topology and temporal evolution signals, rely exclusively on textual similarity to infer linkages, thereby exhibiting an inherent bias toward high recall and low precision that fails to support accurate link prediction.

Benefiting from the spatiotemporal fusion architecture that integrates semantic representations, graph structural topology, and longitudinal evolutionary patterns, the proposed Qwen-STGF achieves comprehensive performance superiority over all Chinese baselines. Qwen-STGF resolves the bottleneck of excessive recall coupled with deficient precision, attaining a precision of 0.8673—a 36.8% relative improvement over the best baseline (ERNIE-3.0-Base-ZH, 0.6342). Although its recall (0.8162) is lower than that of the unconstrained semantic baselines, it establishes a far more balanced and practically viable performance profile. Qwen-STGF delivers an F1-score of 0.8410, marking an 8.8% relative gain over ERNIE-3.0-Base-ZH (0.7729), which validates the efficacy of spatiotemporal feature fusion in enhancing composite prediction quality. Furthermore, Qwen-STGF yields an AUC of 0.8786 and an AUPR of 0.9322, outperforming the leading baseline (Text2Vec-Base-Chinese) by 3.2% and 2.0%, respectively, ranking first across all benchmarked architectures on both ranking metrics.

These empirical findings demonstrate that, compared with standard Chinese text encoding backbones, the Qwen-STGF framework—by virtue of graph structure awareness and temporal trajectory modeling—captures latent associations among academic keywords with markedly higher fidelity, demonstrating superior applicability and effectiveness for link prediction in the Chinese literature knowledge graphs.

#### 3.4.2. Large Model Comparison

One of the core objectives of this study is to develop a widely applicable lightweight scheme for small and medium-sized enterprises (SMEs) and individual researchers with limited large-scale computing resources. Large-parameter models generally require professional cloud-based GPU clusters for deployment, whereas all experiments in this work are implemented solely on a single consumer-grade laptop. Considering the above factors, the Qwen-1.8B-Chat model with 1.8 billion parameters is selected in this research, which can run smoothly on consumer-level hardware.

**Figure 4:**
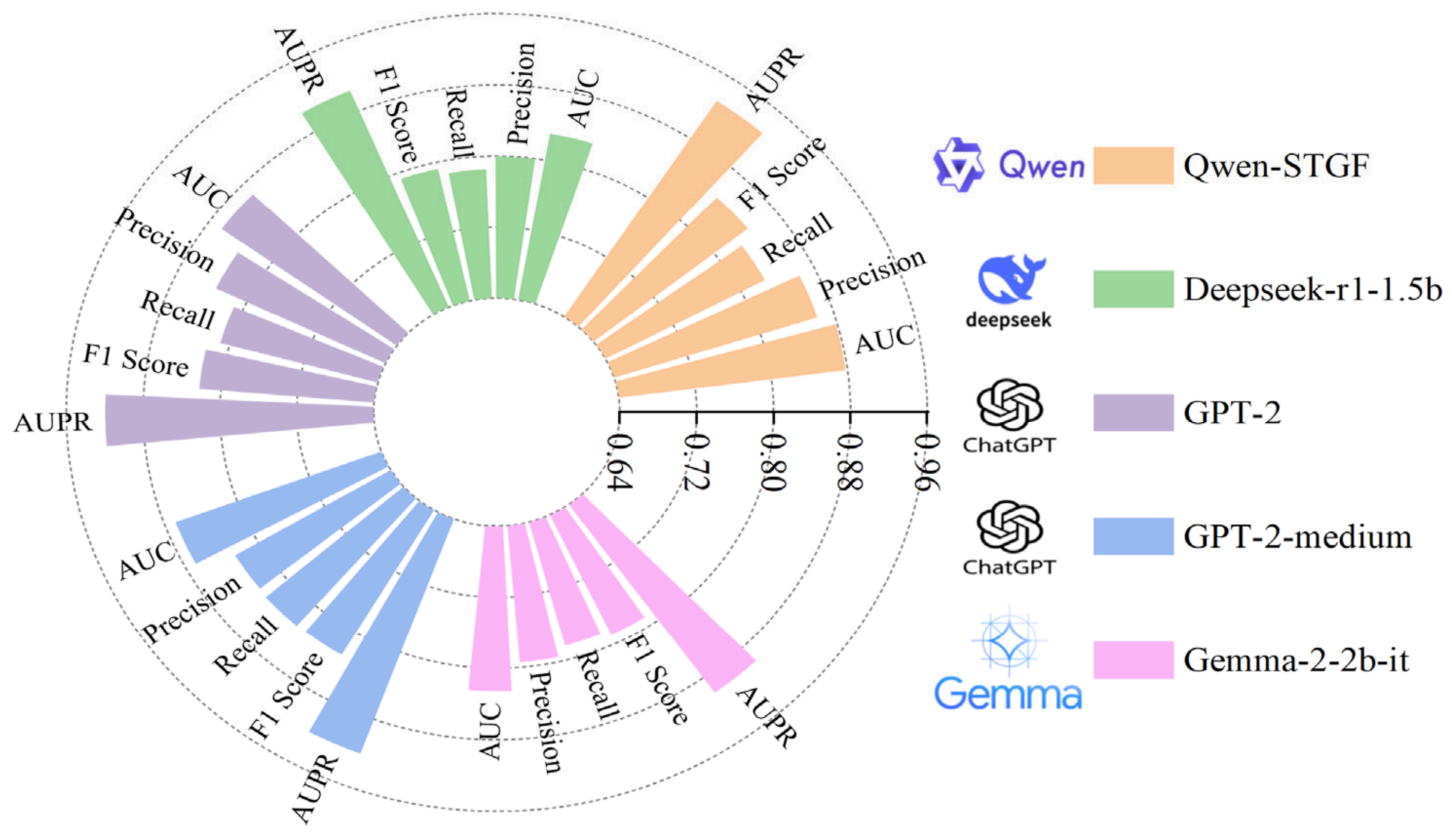
Performance comparison of large models.

To systematically explore the applicability of different large language models of comparable parameter sizes for the given task, four lightweight models with fewer than 3 billion parameters that can run efficiently on consumer-grade hardware are selected for comparative experiments, constrained by the experimental setup consisting of only a single consumer laptop. The detailed configurations of the candidate large models are listed in Table 4, and the corresponding experimental results are presented in Figure 4.

The results are shown in Figure 4. Experiments demonstrate that the Qwen-STGF model ranks first in four core metrics: AUC, Precision, F1-score and AUPR, reaching 0.8786, 0.8673, 0.8410 and 0.9322 respectively. Among them, the F1-score is 1.22 percentage points higher than that of the second-ranked GPT_2_medium. Its recall rate is 0.8298, which is slightly lower than GPT_2_medium by a tiny margin of 0.0136, indicating comprehensive and balanced overall performance.

Comparative analysis results demonstrate that although Gemma-2-2b-it achieves a high AUPR, its semantic representation capability for Chinese domain-specific terminology is constrained by the insufficient adequacy of Chinese corpus pre-training. As a result, its precision and F1-score remain relatively low at 0.7943 and 0.7889 respectively, with drawbacks including a high false positive rate and unsatisfactory comprehensive classification performance. Deepseek-r1-1.5b ranks last across all metrics and exhibits the poorest overall performance. The above findings indicate that among models with comparable parameter scales, Qwen-STGF delivers more accurate semantic representation of domain-specific terminology and disciplinary correlations in the CNKI literature and can more effectively mine deep semantic associations between keywords.

#### 3.4.3. Ablation Experiment Analysis

Ablation variants are constructed to verify the effectiveness of each component within the proposed Qwen-STGF model as described below. The variant without the GraphSAGE module is denoted as STGF-Sage, which deprives the model of the capability to dynamically aggregate structural information of neighboring entities from the keyword co-occurrence network. Since the yearly outputs of GraphSAGE are stacked along the temporal dimension to serve as the input to CNN-LSTM, a pseudo time-series is fed into CNN-LSTM for this ablation setup. Specifically, static textual features extracted by Qwen are duplicated along the time axis to mimic real temporal data.

The variant excluding the CNN-LSTM module is named STGF-Temp, which eliminates the model’s access to historical technological evolution patterns for improving current prediction performance. STGF-SaTe removes both the GraphSAGE and CNN-LSTM modules, whereas STGF-Text ablates the twin-tower semantic module and disables the model from leveraging in-depth semantic information of keywords and titles for performance enhancement. All experimental results are summarized in Table 5.

Through the module removal strategy, the value and complementarity of the GraphSAGE spatial structure module, CNN-LSTM temporal evolution module, and dual-tower semantic module in academic keyword correlation prediction are verified. The results show that the complete Qwen-STGF model achieves the optimal values among all variants on three core metrics (0.8786, 0.8673, 0.9322) for AUC, Precision, and AUPR, realizing the optimal balance between prediction accuracy and generalization ability.

The GraphSAGE module serves as the fundamental support and performance lower-bound guarantee for the overall model. The STGF-Sage variant with this module removed exhibits substantial performance degradation across all evaluation metrics: compared with the full model, its AUC drops by 0.0455, and F1 Score drops by 0.0448. As the ablation group with the largest performance decline, this result indicates that the topological structural information of the keyword co-occurrence network forms the core informational foundation for keyword association prediction. Furthermore, it is verified that the performance gain of the temporal module is highly reliant on the annual dynamic structural features output by GraphSAGE. A significant synergistic effect exists between the two modules, rather than a simple additive performance improvement.

The STGF-Temp and STGF-Text ablation groups exhibit comparable degrees of performance degradation, indicating that the CNN-LSTM temporal module and the Qwen dual-tower semantic module deliver performance gains of the same magnitude. The former improves prediction precision by capturing technological evolution patterns, whereas the latter enhances the model’s generalization capability via deep semantic mining. When integrated with the spatial module, the two modules jointly contribute to the overall performance improvement of the model.

Specifically, the STGF-Text variant completely detaches the dual-tower semantic module (including edge titles); under this configuration, the AUPR only marginally declines from 0.9322 to 0.9235, while the AUC undergoes a minor decrease from 0.8786 to 0.8724. This variant establishes the most rigorous text-free ablation lower bound, corroborating that the primary performance gains stem predominantly from the co-occurrence network topology and temporal dynamics rather than textual cues.

#### 3.4.4. Validation of Prediction Models

To quantitatively validate the reliability of the proposed prediction method, this study conducts historical backtesting using a rolling time window approach. Specifically, word frequency data spanning 2016–2021 serve as the training set to forecast word frequency for the 2022–2025 period. The ARIMA model and Holt’s linear exponential smoothing model are selected as baseline methods for comparison, and mean absolute error (MAE) and root mean square error (RMSE) are adopted as evaluation metrics. The backtesting results are presented in Table 6.

The historical backtesting protocol specifically focused on three core technical keywords: “Road Engineering”, “Asphalt Mixture”, and “Mesostructured”. Deploying a rolling-origin evaluation scheme, the models were trained on longitudinal term frequencies spanning 2016–2021 to perform prospective year-by-year forecasting across 2022–2025 (a 4-year evaluation horizon). Crucially, conventional random cross-validation was strictly circumvented in this temporal backtest to eliminate look-ahead bias and prevent temporal data leakage.

In terms of overall performance, the comprehensive prediction accuracy of the three models presents a distinct hierarchical gradient: the Prophet model delivers the best overall performance, followed by the ARIMA model, while Holt’s linear exponential smoothing model exhibits the poorest overall performance. The Prophet model outperforms the other two models on both evaluation metrics across the two keyword sets, and only slightly lags behind the ARIMA model in extreme error control for high-volatility series.

For individual keywords, the word frequency trend of road engineering is relatively stable. The MAE values of all three models are consistently 2.75, indicating no significant difference in average prediction accuracy. The RMSE values follow the order of Prophet (3.59) < ARIMA (3.71) < Holt (3.89), which reveals that the Prophet model has a more uniform prediction error distribution and superior overall stability.

The longitudinal term frequency sequence of ‘road engineering’ exhibits relatively stable evolutionary dynamics. Across the four evaluation years (2022–2025, *N* = 4), the cumulative sum of absolute deviations between the integer-rounded predictions and authentic frequencies happens to be identically 11 for all three models ($\sum_{t=1}^{4}\left|y_{t}-{\hat{y}}_{t}\right|=11$), naturally resulting in an identical MAE of 2.75 (11/4 = 2.75). This phenomenon represents an arithmetic coincidence arising from discrete integer counts evaluated over a constrained temporal horizon (*N* = 4), rather than a methodological anomaly or reporting error. Conversely, the RMSE—which penalizes single-point extreme deviations quadratically—distinctly differentiates the error variance across the three approaches (Prophet: 3.59 < ARIMA: 3.71 < Holt: 3.89). An identical mathematical rationale applies to the “Asphalt Mixture” series, where both Prophet and ARIMA achieve an identical MAE of 4.25 corresponding to a cumulative absolute deviation of 17 (17/4 = 4.25).

The word frequency of asphalt mixture features the largest fluctuation amplitude. The Prophet and ARIMA models both achieve an MAE of 4.25, showing comparable average prediction accuracy. In terms of RMSE, the ARIMA model reaches 5.45, slightly lower than 6.12 for the Prophet model, suggesting that the ARIMA model has a marginal advantage in mitigating single-point extreme prediction deviations for word frequency series with large magnitudes and high volatility. Both error metrics of the Holt model are nearly twice those of the aforementioned two models, which is attributed to the poor matching between its linear trend extrapolation assumption and the actual fluctuation characteristics of word frequency.

For the keyword mesostructure, the word frequency has a low numerical magnitude and a clear fluctuation pattern, and the Prophet model achieves optimal performance on both metrics. Specifically, its MAE is 2.00, representing a 0.50% reduction compared with the ARIMA model and a 30.31% reduction compared with the Holt model; its RMSE is 2.44, which is 4.69% lower than that of the ARIMA model and 24.22% lower than that of the Holt model. This finding confirms that the Prophet model has more prominent advantages in trend prediction for emerging technology directions such as mesoscopic image processing.

Overall, the Prophet model demonstrates strong adaptability to word frequency series with different fluctuation characteristics and magnitudes. Its advantages in prediction accuracy and stability are particularly prominent for stationary series and low-magnitude word frequency prediction, and its overall generalization ability outperforms the two baseline models. Accordingly, the Prophet model is more suitable for forecasting technology evolution trends in the field of asphalt mixture image processing.

### 3.5. Lightweight Correlation Reasoning and Technology Trend Prediction

To clarify the technological evolution trajectory in the field of asphalt mixture image processing, a corpus is constructed based on time-series keyword data from the CNKI literature database, and a keyword co-occurrence prediction and technology trend forecasting system tailored to the academic field of road engineering is established based on the trained model. Given that the model cannot directly identify potential non-co-occurring keyword pairs, the body of literature highly correlated with the asphalt mixture–image processing technology chain within the corpus, entitled *Review of Research on Digital Twin Models for Asphalt Mixture Performance Based on Mesostructural Characteristics*, is selected as the analytical object for association identification.

#### 3.5.1. Association Reasoning Analysis

The lightweight prediction and identification module functions in two modes. For known vocabularies in the corpus, precomputed feature vectors are directly reused; for unincluded cutting-edge emerging terms and cross-domain unfamiliar vocabularies, a zero-shot recognition mode is activated. Since the input keywords are derived from the known literature, the output keyword association probability is definitively equal to 1.

**Figure 5:**
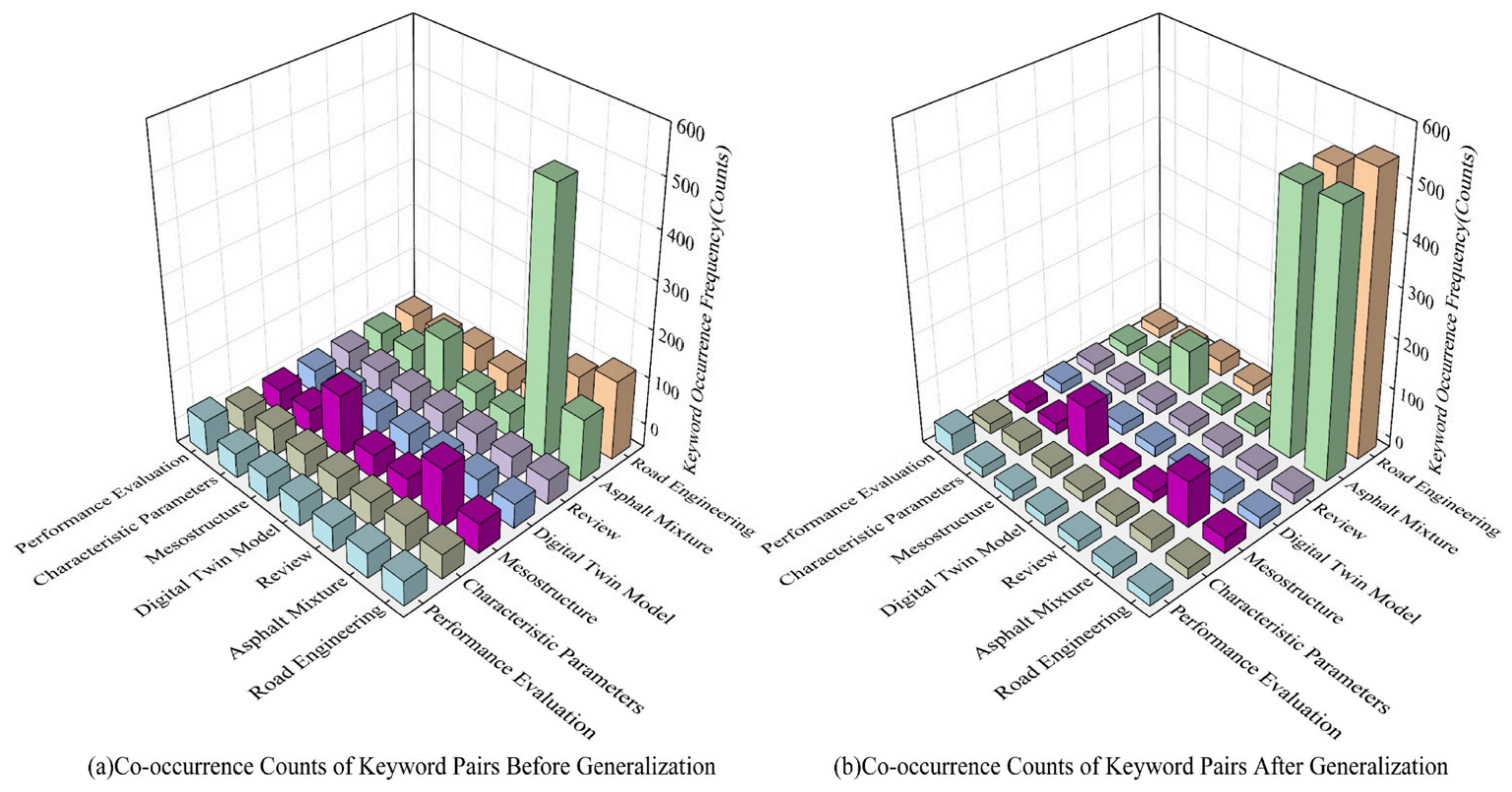
Co-occurrence frequency of keyword pairs.

The model-based association identification analysis can reflect the association strength and integration degree among various research directions along the image processing technology chain. As asphalt mixture falls within the discipline of road engineering, the word frequency of road engineering is inherently higher than that of asphalt mixture. To eliminate the bias induced by the magnitude difference in word frequency, generalization correction is applied to the co-occurrence frequency data, so as to enhance the cross-direction generalization performance of the model. The co-occurrence counts of the seven output keyword pairs before and after generalization are presented in Figure 5.

Taking the target literature as an example, the model classifies keywords in sub-fields such as “warm-mixed recycled fiber asphalt mixture” and “permeable asphalt mixture” into the thematic category of asphalt mixture through semantic clustering.

Among the 768 publications, “road engineering” appears 540 times and “asphalt mixture” appears 509 times, indicating that asphalt mixture is the core research object with the highest proportion in the road engineering literature, as well as the primary application carrier of image processing technology.

“Mesostructure” co-occurs with “asphalt mixture” 72 times, accounting for 88.89% of the total frequency of mesostructure. This result indicates that mesostructure research is highly dependent on the asphalt mixture direction, and mesostructure characterization is precisely the core application scenario of image processing technology in this field. “Mesostructure” appears 81 times, while “performance evaluation” appears only 3 times, with a co-occurrence frequency of merely 1. This finding suggests that most current mesoscopic research based on image processing remains at the morphological observation stage, and no effective structure-performance mapping relationship has been established.

“Characteristic parameters” appear 8 times and co-occur with “mesostructure” 2 times, reflecting that the mesoscopic indicators extracted by image processing have not yet formed a unified characteristic parameter system, which restricts the construction of reliable meso-macro correlation models. The “digital twin model” appears only once and co-occurs with review publications, indicating that the research direction combining digital twins and image processing is still at the concept introduction stage. Based on the above results, the model-based association identification predicts that subsequent research will focus on three major directions: constructing a unified mesoscopic characteristic parameter system, developing dynamic digital twin technology, and establishing a reliable macro-mesoscopic performance correlation model, all of which are deeply coupled with image processing technology.

#### 3.5.2. Trend Prediction Analysis

**Figure 6:**
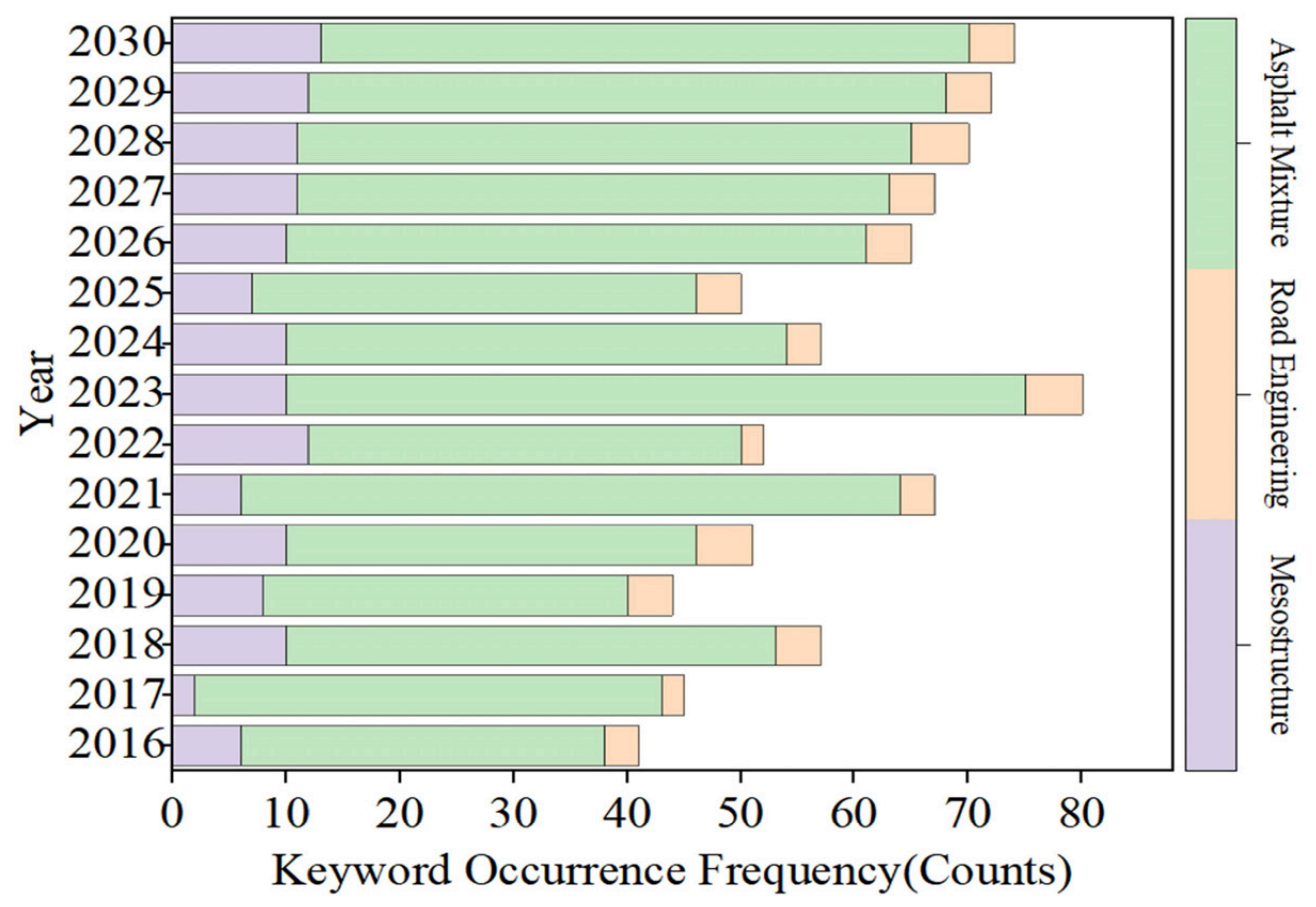
Keyword frequency prediction.

The technology trend prediction module employs the Prophet model [50,51,52], with a trend changepoint prior scale set to 0.05, to perform quantitative prediction of the evolution trends of keywords in the field of asphalt mixture image processing. Given that the four keywords—review, digital twin model, characteristic parameters, and performance evaluation—have excessively low occurrence frequencies (≤3 times), their predictions are prone to distortion. Accordingly, three core keywords that constitute the main research line of asphalt mixture image processing, namely road engineering, asphalt mixture, and mesostructure, are selected for prediction. The keyword frequency predictions are presented in Figure 6.

Prediction trend of road engineering: The frequency increases from 50 occurrences in 2025 to approximately 74 occurrences and maintains a steady upward trajectory during 2026–2030, indicating the continuous expansion of the overall research scale in this field. As a first-level discipline category, its research popularity lays the disciplinary foundation for the development of interdisciplinary technologies such as asphalt mixture image processing and digital twins. The deep integration of image processing and road engineering will continuously give rise to new research directions, providing engineering application scenarios for asphalt mixture digital twin technology.

Prediction trend of asphalt mixture: The frequency recovers steadily from 46 occurrences in 2025, reaches 61 occurrences in 2026, and then gradually increases to approximately 70 occurrences by 2030. The growth rate is moderate (14.75% over five years), and the overall level remains stable at a high plateau. Over the next five years, asphalt mixture will remain the core carrier of image processing research in road engineering, and fundamental research conducted around it—including image segmentation, mesoscopic identification, and feature quantification—will continue to dominate the field.

Prediction trend of mesostructure: The frequency increases gradually from 7 occurrences in 2025 to 13 occurrences in 2030, registering the fastest five-year growth rate among the three keywords. It exhibits a linear growth trend with no obvious signs of slowdown, indicating that image-processing-based mesostructure characterization will become the sub-direction with the greatest growth potential in the road engineering field over the next five years. From a technical logic perspective, the accurate, real-time, and quantitative acquisition of mesostructure will directly drive the rapid implementation of cutting-edge research areas such as dynamic digital twin updating, multi-scale data fusion, and structure–performance correlation modeling.

Integrating large model-based association identification and trend prediction analysis, this study identifies the research bottlenecks in the field of asphalt mixture image processing and predicts three major future research directions: constructing a unified mesoscopic characteristic parameter system, developing dynamic digital twin technology, and establishing a reliable macro-mesoscopic performance correlation model. This study reveals a quantifiable mapping relationship between the semantic features of academic keywords and the technology evolution trends in the interdisciplinary field of road engineering and computer vision (image processing): the co-occurrence frequency of keywords corresponds to the maturity of technology integration, while the predicted trend of keywords corresponds to the explosive potential of technology. The future technology development directions derived from this mapping relationship can effectively circumvent the subjectivity of traditional expert judgment, thereby providing an objective and quantitative theoretical reference for research topic selection, technology iteration, and key research priorities in the field of asphalt mixture image processing.

### 3.6. Discussion

This study is positioned within the burgeoning paradigm of materials informatics and literature-based discovery (LBD). Rather than conducting redundant physical trials on asphalt mixtures, the primary objective is to develop a lightweight, data-driven computational compass that infers cross-domain technological associations and anticipates evolutionary trajectories, thereby offering prospective navigational guidance for experimental road materials research. The empirical validation encompasses 768 screened publications, 1903 keyword nodes, and 13,915 annotated edges, benchmarked systematically across conventional machine learning baselines, graph neural networks, native Chinese baseline models, and open-source LLMs of comparable scale, supplemented by rigorous ablation studies and rolling-origin temporal backtesting. Compared with existing materials informatics frameworks, the hallmark of this work lies in achieving high-fidelity predictive modeling under consumer-grade hardware constraints through a collaborative semantic–spatial–temporal fused representation.

The three prospective technological trajectories forecasted by the model exhibit direct correspondence with verifiable empirical frequency evidence from the corpus: “Performance Evaluation” appears merely 3 times and co-occurs with “Mesostructured” only once, underscoring that current image-based mesoscopic investigations remain largely confined to qualitative morphological observations (e.g., X-ray micro-computed tomography or 2D optical cross-sections) without establishing robust structure-property mappings to macroscopic mechanical performance metrics (such as Hamburg Wheel Tracking for rutting resistance or semicircular bending for fracture toughness). Furthermore, “Digital Twin Model” registers merely a single occurrence, reflecting an embryonic conceptual introduction stage, whereas 88.89% of all mesostructural investigations are intrinsically anchored to asphalt mixtures. These insights strictly align with established consensus in pavement materials science and are fully verifiable against the corpus, rather than being subjective hallucinations of the model.

From an architectural standpoint, the proposed Qwen-STGF framework is intrinsically material-agnostic: the twin-tower semantic backbone, dynamic co-occurrence graph modeling, CNN-LSTM temporal tracking, multimodal fusion, and cost-sensitive classification incorporate no asphalt-specific physical heuristics, with the literature query formulation and text corpus serving as the sole domain-dependent inputs. When transferred to other advanced materials (e.g., fiber-reinforced cementitious composites, solid-state battery electrolytes, or additive manufacturing alloys) and diagnostic, testing, or numerical modeling methodologies, researchers can follow a standardized five-step transfer pipeline: (1) query formulation; (2) corpus curation with unified filtering; (3) keyword tokenization, topological edge assembly, and controlled negative sampling; (4) execution of the phased multimodal training pipeline; and (5) inference of prospective associations and temporal trends, without requiring structural modifications to the model architecture. While author-curated titles and keywords offer low noise and universal cross-database accessibility ideal for lightweight deployment, they inherently exhibit granularity constraints and are presently restricted to a single-source Chinese repository. Future extensions will integrate publication abstracts, experimental methodology sections, and comprehensive multilingual repositories (including Web of Science, EI, and Scopus) to capture granular testing protocols and scanning resolutions, thereby bolstering cross-material generalizability.

## 4. Conclusions

(1) An integrated research system for intelligent literature mining and trend analysis tailored to the field of asphalt mixture image processing is constructed. A full-process solution is established, encompassing literature data preprocessing, training of the semantic–spatial–temporal three-dimensional fusion feature model, lightweight online identification, and technology trend assessment, which effectively addresses the limitations of traditional literature analysis methods, such as single feature dimension and the difficulty of simultaneously accommodating association prediction and temporal deduction.

(2) The Qwen-STGF model demonstrates favorable feature fusion capability and comprehensive prediction performance. Comparative experiments are conducted with multiple traditional machine learning models, classical graph neural network models, and open-source large models of comparable scale. The results indicate that the Qwen-STGF model achieves optimal values on all three core evaluation metrics—AUC, F1-score, and AUPR—reaching 0.8786, 0.841, and 0.9322, respectively, while maintaining high levels of precision and recall. The model effectively integrates multi-dimensional features of semantics, space, and time, achieving a sound balance between prediction accuracy and generalization ability.

(3) A quantitative correlation between the features of academic keywords and the technology evolution trends in the interdisciplinary field of road engineering and computer vision (image processing) is preliminarily revealed: the co-occurrence frequency of keywords can, to a certain extent, reflect the maturity of technology integration, while the predicted trend of keywords can indicate the potential explosive potential of technology. Accordingly, three major future research directions are predicted: constructing a unified mesoscopic characteristic parameter system, developing dynamic digital twin technology, and establishing a reliable macro-mesoscopic performance correlation model, all of which serve as technical support for asphalt mixture image processing.

This study still has certain limitations. First, the current research only utilizes the titles, keywords, and publication years of the literature, without incorporating abstracts and full-text content, which may result in the loss of some detailed technical information and research progress. Future research will expand the input data scope of the framework to include the literature abstracts and full-text content, thereby extracting finer-grained technical features and research progress from the full text.

## Figures and Tables

**Table 1:** Formulas of evaluation metrics.

Evaluation Metrics	Formula
AUC [37]	$\mathrm{AUC}=\sum_{i=1}^{n-1}\frac{\left({\mathrm{FPR}}_{i+1}-{\mathrm{FPR}}_{i})\cdot ({\mathrm{TPR}}_{i+1}+{\mathrm{TPR}}_{i}\right)}{2}$, $\mathrm{FPR}=\frac{\mathrm{FP}}{\mathrm{FP}+\mathrm{TN}}$, $\mathrm{TPR}=\frac{\mathrm{TP}}{\mathrm{TP}+\mathrm{FN}}$
Precision [38]	$\mathrm{Precision}=\frac{\mathrm{TP}}{\mathrm{TP}+\mathrm{FP}}$
Recall [39]	$\mathrm{Recall}=\frac{\mathrm{TP}}{\mathrm{TP}+\mathrm{FN}}$
F1 Score [40]	$F1\;\mathrm{Score}=2\times \frac{\mathrm{Precision}\cdot \mathrm{Recall}}{\mathrm{Precision}+\mathrm{Recall}}$
AUPR [41]	$\mathrm{AUPR}=\sum_{i=1}^{n-1}\frac{\left(R_{i+1}-R_{i})\cdot (P_{i+1}+P_{i}\right)}{2}$

where *FPR_i_* is the false positive rate for the *i*-th sample; *TPR_i_* is the true positive rate for the *i*-th sample; TP denotes the number of true positives correctly predicted by the model; FP denotes the number of false positives incorrectly predicted by the model; TN denotes the number of true negatives correctly predicted by the model; FN denotes the number of false negatives incorrectly predicted by the model.

**Table 2:** Different parameter configurations.

Model	Negative Sample Edge Ratio	Keyword Weight	Title Weight	Epochs	Contrastive Loss
M1	0.8	3.4	2.9	9	0.045
M2	0.8	3.8	2.9	9	0.045
M3	0.8	3.6	2.9	9	0.045
M4	0.8	3.6	3.1	9	0.045
M5	0.8	3.6	3	9	0.045
M6	0.8	3.6	2.95	9	0.045
M7	0.65	3.6	2.95	9	0.045
M8	0.63	3.6	2.95	9	0.045
M9	0.62	3.6	2.95	9	0.045
M10	0.625	3.6	2.95	9	0.04
M11	0.625	3.6	2.95	9	0.05
M12	0.625	3.6	2.95	10	0.045
M13	0.625	3.6	2.95	8	0.05
M14	0.625	3.6	2.95	8	0.045
M15	0.625	3.6	2.95	9	0.045

**Table 3:** Chinese model performance comparison.

Method	AUC	Precision	Recall	F1 Score	AUPR
BERT-base-Chinese	0.8406	0.6291	0.9931	0.7703	0.9081
Chinese-RoBERTa-wwm-ext	0.8406	0.6254	0.9958	0.7683	0.9074
ERNIE-3.0-Base-ZH	0.8323	0.6342	0.9894	0.7729	0.9030
BGE-small-zh-v1.5	0.8344	0.6243	0.9956	0.7674	0.9045
Text2Vec-Base-Chinese	0.8515	0.6178	0.9992	0.7635	0.9142
Qwen-STGF	0.8786	0.8673	0.8162	0.8410	0.9322

**Table 4:** Summary of large model parameters.

Model	Abbreviations Used in This Paper	Provider	Parameter Scale	Context Window	Release Time
DeepSeek-R1-Distill-Qwen-1.5B	Deepseek-r1-1.5b	DeepSeek	1.54B	128K tokens	20 January 2025
GPT-2	GPT-2	OpenAI	124M	1024 tokens	14 February 2019
GPT-2 Medium	GPT-2-medium	OpenAI	355M	1024 tokens	14 February 2019
Gemma-2-2b-it	Gemma-2-2b-it	Google	2.6B	8192 tokens	31 July 2024
Qwen-1.8B-Chat	Qwen-STGF	Alibaba Cloud	1.8B	8192 tokens	30 November 2023

**Table 5:** Comparison results of ablation experiments.

Method	AUC	Precision	Recall	F1 Score	AUPR
STGF-Sage	0.8331	0.7813	0.8115	0.7962	0.8870
STGF-Temp	0.8752	0.8301	0.8458	0.8379	0.9244
STGF-SaTe	0.8671	0.7837	0.9114	0.8427	0.9056
STGF-Text	0.8724	0.8531	0.8180	0.8352	0.9235
Qwen-STGF	0.8786	0.8673	0.8162	0.8410	0.9322

**Table 6:** Backtest error comparison of different forecasting methods.

Method	Metrics	Prophet	ARIMA	Holt
Road Engineering	MAE	2.75	2.75	2.75
	RMSE	3.59	3.71	3.89
Asphalt Mixture	MAE	4.25	4.25	8.32
	RMSE	6.12	5.45	9.54
Mesostructure	MAE	2.00	2.01	2.87
	RMSE	2.44	2.56	3.22

## Data Availability

The data used to support the findings of this study are available from the corresponding author upon reasonable request.

## Abbreviations

The following abbreviations are used in this manuscript:

Qwen-STGF	Qwen-based Semantic–Temporal–Graph Fusion
GraphSAGE	Graph Sample and Aggregate
CNN-LSTM	Convolutional Neural Network-Long Short-Term Memory
NLP	Natural Language Processing
SMEs	small and medium-sized enterprises
MSE	mean squared error
CNKI	China National Knowledge Infrastructure
AUC	Area Under the Receiver Operating Characteristic Curve
AUPR	Area Under the Precision–Recall Curve
LogR	Logistic Regression
SVM	Support Vector Machine
RF	Random Forest
XGBoost	eXtreme Gradient Boosting
AdaBoost	Adaptive Boosting
GAT	Graph Attention Network
GCN	Graph Convolutional Network
